# Correction: Niche overlap across landscape variability in summer between two large herbivores using eDNA metabarcoding

**DOI:** 10.1371/journal.pone.0306938

**Published:** 2024-07-05

**Authors:** Eduard Mas-Carrió, Marcin Churski, Dries Kuijper, Luca Fumagalli

In Fig 1C, the color coding for herbispaces is incorrect. Please see the correct Fig 1 here.

**Fig 1:**
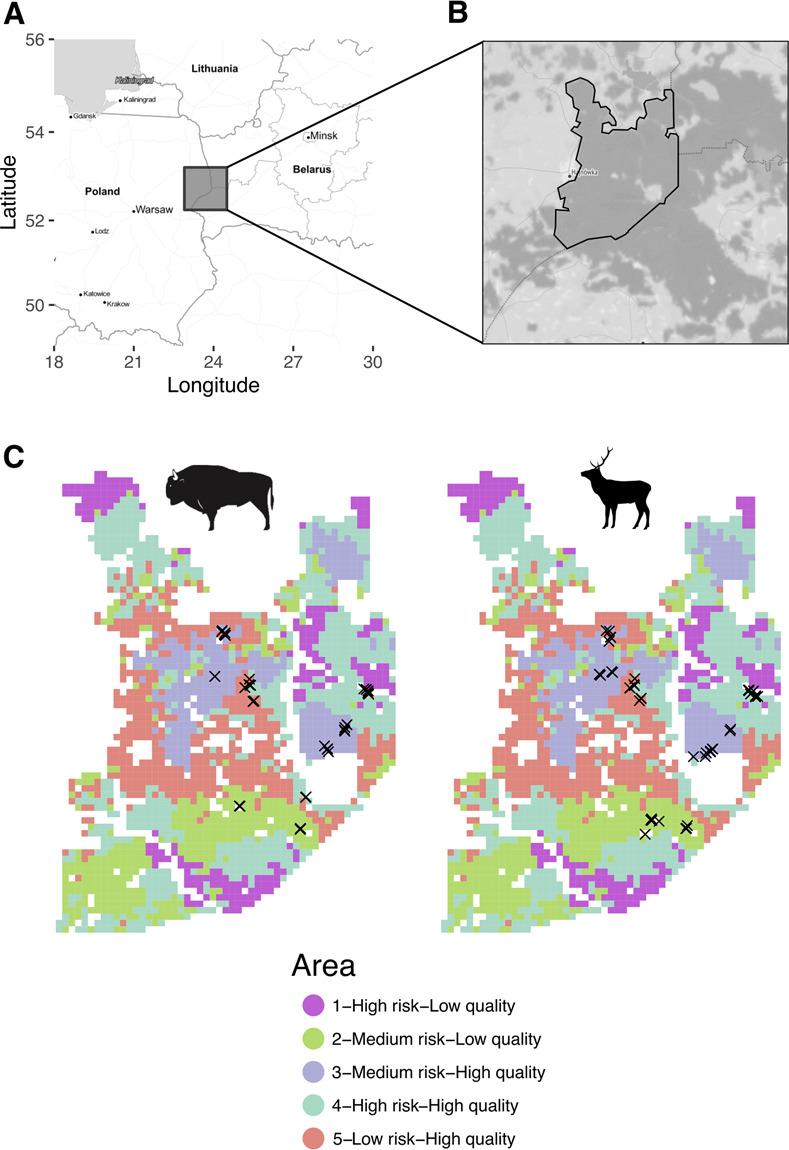
Maps of the study area. A) Large scale map of the area. Grey square marks the area where the Bialowieza forest is located. B) Dark grey areas indicate forested areas. Full black lines mark the area of study within the Bialowieza forest. Dashed black line indicates the limits of the Bialowieza National Park. Notice the eastern edge of the study area is limited by the border with Belarus. C) Area of study divided by the different herbiscape described in Bubnicki et al. 2019. We used the same herbiscape numbering. Each herbiscape has a unique and arbitrary color (herbiscape numbering is maintained as in [21]). Crosses indicate the location where each scat sample was collected for each species. Map tiles used in A) and B) were extracted from Stamen Design, under CC BY 4.0. Data by OpenStreetMap, under ODbL.
