# Supplementary material for: Niche overlap across landscape variability in summer between two large herbivores using eDNA metabarcoding
Source: PLoS One. 2024 Feb 13;19(2):e0279672. doi: 10.1371/journal.pone.0279672 (PMC10863879; doi:10.1371/journal.pone.0279672)
Supplement: S2 File — (DOCX) [file pone.0279672.s002.docx]

### **Supplementary material**

*DNA amplification and library preparation*

qPCR reagents and conditions were the same as in DNA metabarcoding PCR reactions (see below), with the addition of 10,000-fold diluted SybrGreen (Thermo Fisher Scientific, USA).

To assign the DNA sequences to each sample, primers were tagged with eight variable nucleotides added to their 5’-end with at least five differences between tags. Tags also included 2 to 4 random nucleotides on the tag 5'-end to increase variability and improve sequencing performance. The PCR reactions were performed in a final volume of 20 µL. The mixture contained 1 U AmpliTaq® Gold 360 mix (Thermo Fisher Scientific, USA), 0.04 µg of bovine serum albumin (Roche Diagnostics, Basel, Switzerland), 0.2 µM of tagged forward and reverse primers and 2 µL of 5-fold diluted template DNA. PCR cycling conditions were denaturation for 10 minutes at 95 °C, followed by 40 cycles of 30 s at 95 °C, 30 s at 52 °C and 1 min at 72 °C, with a final elongation step of 7 min at 72 °C. Amplifications were performed separately for each species and in replicates (3 per sample divided in 6 plates, 3 for each species) in PCR plates with controls. Amplicons were purified using a MinElute PCR Purification Kit (Qiagen, Hilden, Germany). Purified pools were quantified using a Qubit® 2.0 Fluorometer (Life Technology Corporation, USA).

Plates contained 50 DNA extracts, 11 blanks as well as 8 extraction, 8 negative and 8 positive PCR controls (DNA assembly of 10 species with increasing relative concentrations). The use of blanks allows estimating the proportion of tag switches (i.e., false combination of tags, generating chimeric sequences) during library preparation (Schnell et al. 2015). Amplification success and fragment sizes were confirmed on a 1.5 % agarose gel. PCR products were subsequently pooled per PCR plate.

Library preparation was done following the recently published TagSteady Protocol (Carøe & Bohmann 2020). After adapter ligation, libraries were validated on a fragment analyzer (Advanced Analytical Technologies, USA).

*Sequence filtering using obitools*

Forward and reverse reads were assembled with a minimum quality score of 40. The joined sequences were assigned to samples based on unique tags combinations. Assigned sequences were then de-replicated, retaining only unique sequences. All sequences with less than 100 reads per library were discarded as well as those not fitting the range of metabarcode lengths. This was followed by two different clustering methods. First, pairwise dissimilarities between reads were computed and lesser abundant sequences with single nucleotide dissimilarity were clustered into the most abundant ones. Second, we used the *Sumaclust* algorithm (Mercier et al. 2013) to further refine the resulting clusters based on a sequence similarity of 97 %. It uses the same clustering algorithm as UCLUST (Prasad et al. 2015) and it is mainly used to identify erroneous sequences produced during amplification and sequencing, derived from its main (centroid) sequence.

*Sequence filtering using metabaR*

Sequences that were more abundant in extraction and PCR controls than in samples were considered as contamination and removed. Operational taxonomic units (OTUs) with similarity to the reference sequence lower than 97 % were also eliminated from the dataset. Removal of tag-leaked sequences was done independently for each library. This approach allowed us to discard single OTUs instead of whole PCR replicates. However, PCR replicates with too small reads count were also discarded.

### **Supplementary figures**


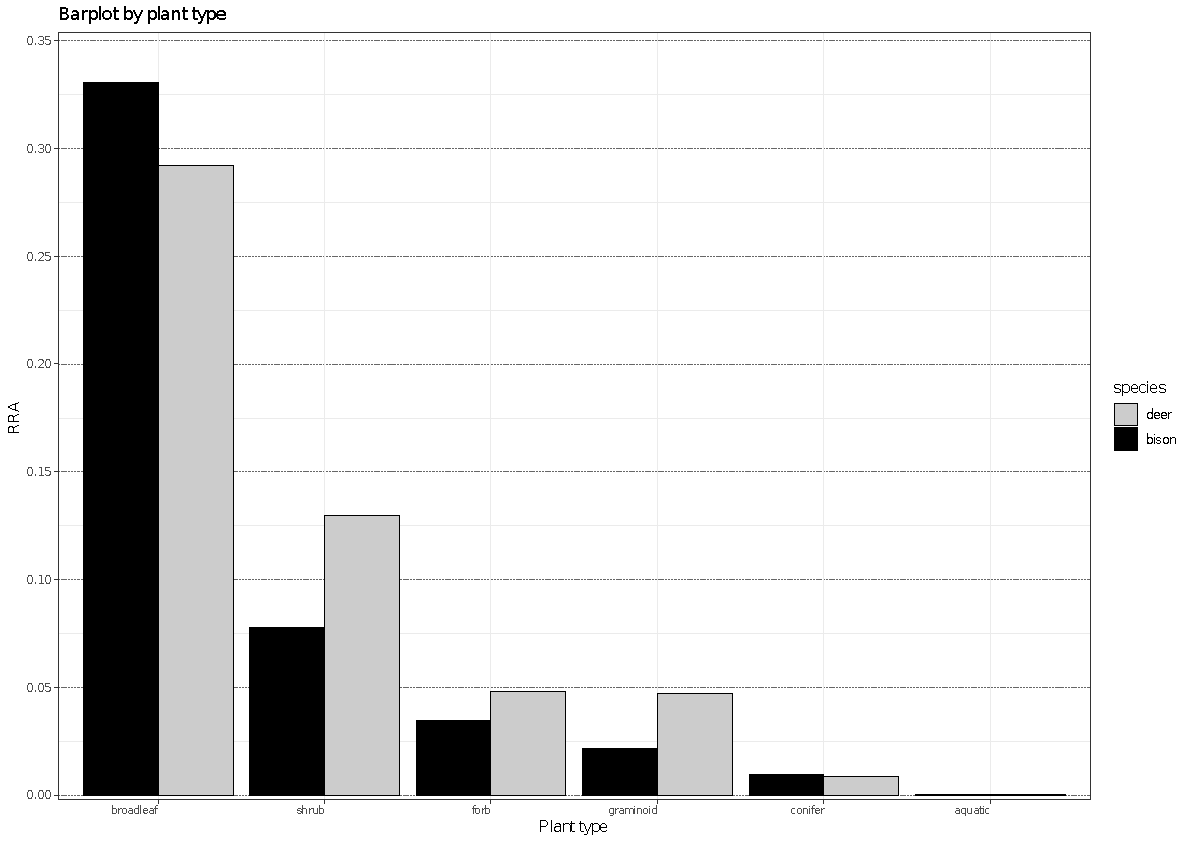


*Supplementary Figure 1 - Barplot by plant type and species, all individuals summed.*


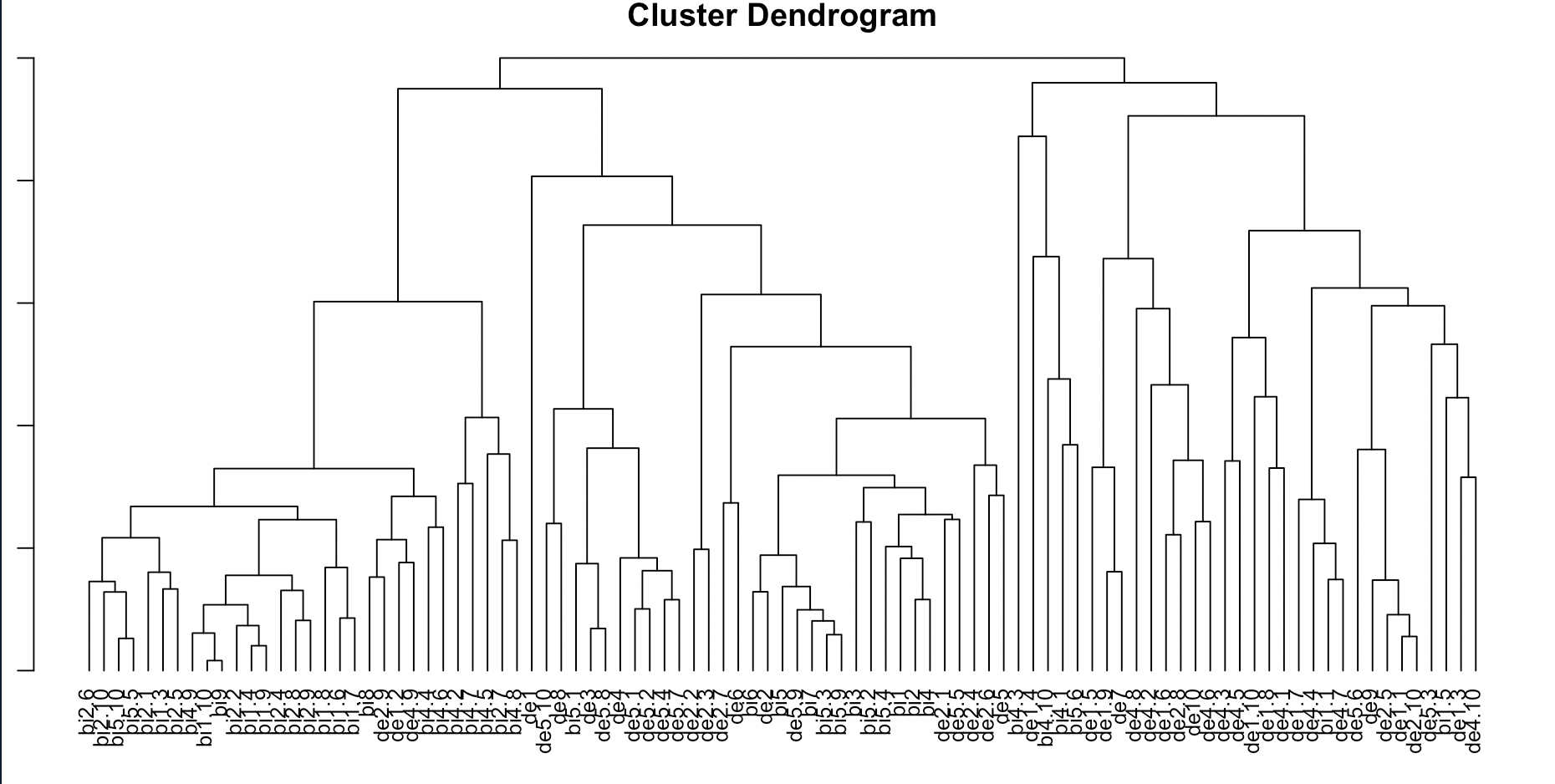


*Supplementary Figure 2 - Cluster dendrogram of diets (de = deer, bi = bison). Clustering analysis group species by diet composition similarity in a tree-like visualisation. If the first split of branches is grouped by species, all diets within species are more similar between each other than to any of the other species' individual diets.*


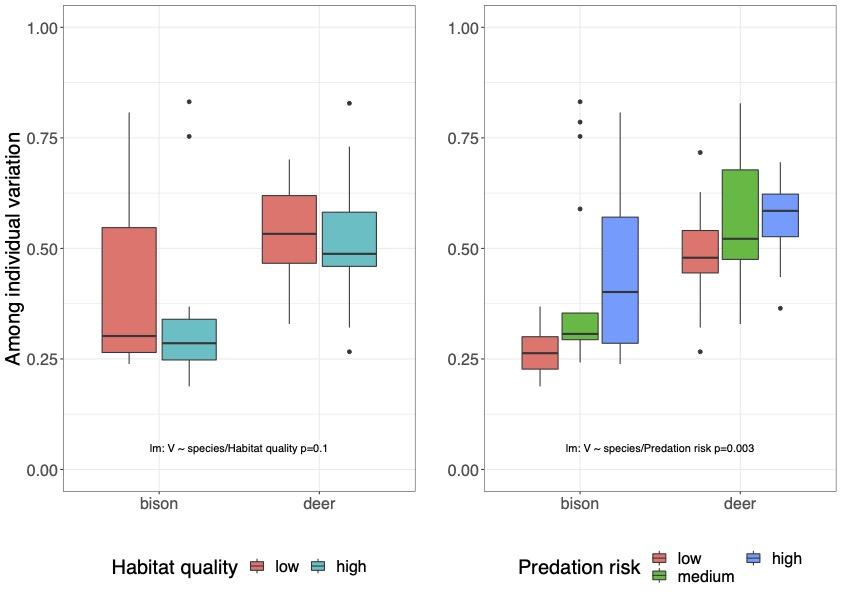


*Supplementary Figure 3 - Among individual diet variation modelling against the two studied environmental factors, i.e., habitat quality (left) and predation risk (right).*
