# Supplementary figures and images for: Niche overlap across landscape variability in summer between two large herbivores using eDNA metabarcoding
Source: PLoS One. 2024 Feb 13;19(2):e0279672. doi: 10.1371/journal.pone.0279672 (PMC10863879; doi:10.1371/journal.pone.0279672)

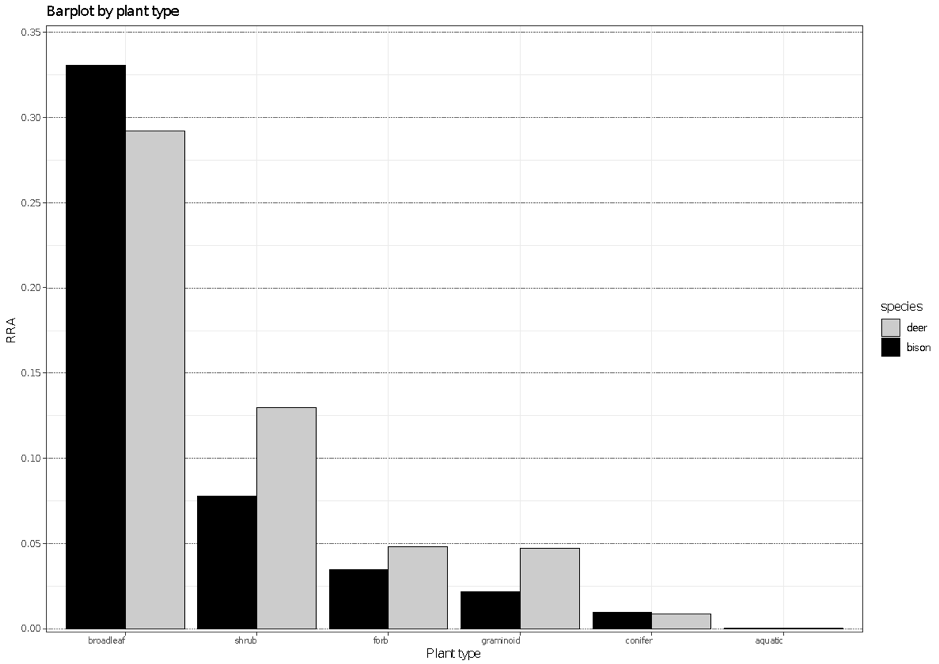

Supplement: S1 Fig — Barplot by plant type and species, all individuals summed. (PNG) [file pone.0279672.s003.png]

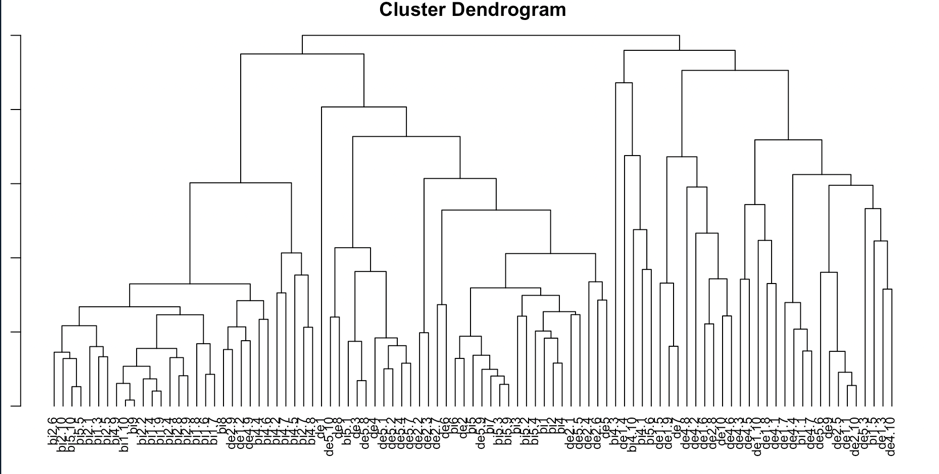

Supplement: S2 Fig — Cluster dendrogram of diets (de = deer, bi = bison). Clustering analysis group species by diet composition similarity in a tree-like visualisation. If the first split of branches is grouped by species, all diets within species are more similar between each other than to any of the other species’ individual diets. (PNG) [file pone.0279672.s004.png]
